# The association between socioeconomic status perception and mental health among Chinese older adults: the mediating roles of social trust and justice

**DOI:** 10.1186/s12877-024-04968-5

**Published:** 2024-06-06

**Authors:** Xiaoxing Ran, Xiaodong Zhang, Wenyi Gong, Gong Chen

**Affiliations:** 1 Institute of Population Research, Peking University, 100871 Beijing, China; 2 Oxford Institute of Population Ageing, University of Oxford, OX2 6PR Oxford, UK

**Keywords:** SES perception, Mental health, Chinese older adults, Social trust, Social justice

## Abstract

**Background:**

Mental health is a matter of quality of life among older adults. This study aimed to explore the association between the socioeconomic status (SES) perception and mental health of older adults using data from 2017 Chinese General Social Survey (CGSS).

**Methods:**

Ordinary least squares (OLS) regression was used to analyse the association between SES perception and mental health, and the substitution model and variable methods were used to check the robustness of the results. Moreover, we adopted the Sobel model to analyse the mediating roles of social trust and justice.

**Results:**

SES perception was positively associated with mental health, and this association was mediated by social trust and justice. This kind of positive association was mainly embodied in those groups with the highest or lowest objective SES. In other words, this study confirmed the phenomenon of “*a contented mind is a perpetual feast*” in Chinese society.

**Conclusions:**

Higher SES perception is associated with improved mental health for Chinese older adults. It is imperative to prioritize efforts to enhance the perceptual abilities of older adults, particularly those with the highest or lowest objective SES, to promote their overall subjective well-being.

## Introduction

The pace of population ageing in China is accelerating at an unprecedented rate. Data from the National Bureau of Statistics showed that the population aged 60 years and older was 296.97 million in 2023, accounting for 21.1% of the total population. The mental health of older adults is crucial for maintaining a good quality of life. However, due to loneliness, low economic status, physical health issues, etc., an increasing number of older adults are at risk of poor mental health. Mental health is related to the overall quality of life of older individuals. Therefore, improving the mental health of older adults has become an urgent priority.

Numerous studies have demonstrated a strong positive relationship between socioeconomic status (SES) and health [1–7]. A low socioeconomic level is known to be linked to higher odds of depression [8] and other mental problems [9]. Previous studies mainly divided SES into two dimensions: subjective and objective [10]. Subjective SES is a more nuanced measure [11–13], while it is less known than objective indicators [10]. One subjective measurement of socioeconomic traits is perceived status, which represents cognitive averaging [13, 14]. This concept has been widely defined as subjective SES [7, 15], perceived social position [14], and SES perception [16]. In this study, we adopt the term “SES perception” to primarily emphasize perceptual ability. It reflects an individual’s subjective assessment of their objective SES.

Existing studies have mainly analysed the determinants of mental health based on objective indicators of SES to explore its relationship with mental health [17–19], and subjective SES has been greatly neglected. SES perception represents an individual’s relative position in society [15] and can be regarded as a subjective indicator of SES. It has been proven that higher objective SES may enhance the mental health of older adults through health-related resources. However, the relationship between SES perception and mental health among older adults, as well as the underlying mechanisms that produce this association, remain unclear. The main aims of this study are to explore the relationship between SES perception and the mental health of Chinese older adults and to further examine the potential mediating mechanism.

## Literature review and hypotheses

### The relationship between SES perception and mental health

Disparities in SES contribute to creating a health gradient [3]. The three main determinants of health—health care, environmental exposure, and health behavior—are rooted in SES [20]. Since SES perception is an overall reflection of objective SES, individuals with higher SES perception often experience better living conditions, interpersonal relationships, and greater access to healthcare services, which could reduce their health risks [3, 21, 22]. People with low SES perceptions have restricted access to material resources, including wealth, income, and level of education [23], which may lead to more exposure to health risks and fewer resources to cope with those challenges [24].

According to relative deprivation theory, individual’s opinion and emotions are influenced by comparing themselves with others who are more fortunate [25]. This can lead to feelings of injustice and annoyance when they perceive others as wealthier [26]. Therefore, people with low SES perceptions are more unhealthy due to the negative sentiment [7, 27–29]. Current disadvantages or negative experiences in childhood are strongly associated with poorer mental health [8, 9, 30–32]. Nevertheless, limited studies have proven that lower SES perception is connected to mental health in different groups, including children [10], teenagers [9, 33], immigrants aged 25 and above [34], and Mexican-origin individuals [35]. We argue that similar results may apply to older adults in China.

Hence, we propose research **hypothesis 1**: SES perception is positively associated with mental health among Chinese older adults.

### The mediating roles of social trust and justice

Beyond direct relationships, the indirect association between SES and the mental health of older individuals has also been confirmed [29], with social trust as one critical intermediary. The environment, crucial in shaping people’s upbringing and life experiences, exerts a profound and enduring influence on their individual and societal identities. This, in turn, significantly affects their mental and emotional responses to their social surroundings [36]. Specifically, differences in individuals’ SES perceptions may alter their attitudes towards society, which constitute a holistic response system [37]. In this context, economic inequality can erode an individual’s trust [38], and SES perception is positively associated with social trust [39–41], suggesting that enhancing SES perception could lower the costs related to fostering social trust.

In addition, social trust is intricately related to mental health [42, 43]. Advancement of social trust can lead to enhanced health outcomes [24]. Research has confirmed a negative correlation between depressive symptoms and social trust [44]. Differences in social trust tied to SES and further exacerbated health inequities, particularly concerning the acquisition of resources [45]. Furthermore, the initial level and increasing rate of social trust are crucial mechanisms through which SES perception indirectly impacts self-rated health [46]. Therefore, social trust could be seen as a mediating variable in the relationship between SES perception and health [47].

Hence, we propose research **hypothesis 2**: Social trust plays a mediating role in the relationship between SES perception and mental health.

Social justice in this study refers to an individual’s perception of social justice. It is an overall perception of social fairness [48]. To compare their condition to other possibilities, individuals utilize a principle that defines what ‘ought to be’ [49]. The higher the SES perception, the more positively the society is evaluated, so the sense of social justice is significantly affected by SES perception [50]. Some scholars found that lower SES perceptions lead to a lower perception of social equity [51]. For each level of SES perception, individuals’ perception of social justice increased by 66.7% [52]. In addition, a sense of social justice can alleviate mental problems [53], while a sense of discrimination can undermine mental health [54]. Furthermore, in terms of psychological paradigms, individuals who experience unfairness in society are more likely to experience anxiety [55], which is detrimental to their mental health. We argue that the sense of social justice may play a mediating role [50].

Therefore, we propose **hypothesis 3**: Social justice plays a mediating role in the relationship between SES perception and mental health.

### A contented mind is a perpetual feast: the relationship between SES perception and the mental health of older adults under the same objective SES

Individuals’ SES perceptions are influenced by personal and social factors [56]. People’s perceptions are rooted in their unique life experiences, reflecting their objective SES [57]. Different objective SES cause various life experiences and subjective evaluations of individuals’ SES [55]. Moreover, SES perception is also influenced by other factors. For instance, it may be affected by elements of their local environment, which determines the persons with whom they compare their status [58]. As a consequence, they rate themselves concerning others based on their perceived social status [59]. Therefore, various groups may have different SES perceptions, although they may appear under the same objective SES. That is, the SES perception of an individual may not be compatible with his or her objective SES [12, 60]. Comparing oneself to those with lower SES tends to increase one’s SES perception, while comparing oneself to those with higher SES generally decreases it [61]. To clarify the role of SES perception, this article separated older adults into several groups according to objective SES and examined whether the phenomenon of *“a contented mind is a perpetual feast*” exists in Chinese society.

We propose **hypothesis 4**. Under the same objective SES, the higher the subjective SES perception, the better the mental health of Chinese older adults.

## Methods

### Data source

The data we used in this study were from the 2017 Chinese General Social Survey (CGSS), conducted by National Survey Research Center of Renmin University of China. The CGSS is the first nationwide, comprehensive, and continuous academic survey initiative in China and covers 31 provinces/autonomous regions/municipalities in mainland China. The stratified and multistage probability sampling method was adopted to select the sample. The questionnaire mainly includes 3 modules: the Core module, the Social Networks and Network Society module, and the Household Questionnaire module. These modules can comprehensively collect demographic, SES perception, health status, social trust and justice, and other information from Chinese adults.

The 2017 CGSS consisted of 12,582 valid samples. According to research demands, we cleaned the data as follows. First, since the research targets in this study were older adults, we only kept those samples aged 60 and over. Moreover, we deleted the missing values of all essential variables and extreme values of income. A final sample size of 4045 was obtained, and we used these samples to analyse the relationship between SES perception and mental health of Chinese older adults.

### Measures

#### Dependent variable: mental health

The dependent variable was the mental health of Chinese older adults. Referring to previous studies [62, 63] and questionnaire design, we used the question “In the past four weeks, how often have you felt depressed or frustrated?” to measure the mental health status of older adults. The options are “Always, Often, Sometimes, Rarely, and Never”. We assigned a value of 1 to 5 if the respondent chose “Always, Often, Sometimes, Rarely, and Never”, respectively.

#### Independent variable: SES perception

SES perception has been regarded as a subjective assessment indicator of relative SES [15]. According to previous studies [7, 40, 50, 52], we chose the question “Overall, where do you personally fall in terms of SES in the current society?”. Respondents were required to make a choice from 5 options (1 = lower, 2 = lower-middle, 3 = middle, 4 = upper-middle and 5 = upper). Moreover, we used another question: “In current society, which social class do you personally remain in? (social class varies from ‘1’ to ‘10’)” to check the robustness of the regression results. ‘1’ represented the lowest SES perception. Conversely, ‘10’ represented the highest SES perception [12, 34].

#### Mediating variables: social trust and justice

In this study, we used social trust and justice as the mediating variables. The sense of social trust was measured by the question ‘Overall, do you agree that the majority of people in this society can be trusted?’, and the following options were included: ‘1 = Strongly disagree, 2 = Relatively disagree, 3 = Cannot say I agree or disagree, 4 = Relatively agree, and 5 = Strongly agree’. Higher levels of agreement represent higher levels of social trust [64]. Similarly, the sense of social justice was measured by the question “In general, do you think current society is fair or unfair?”. There are five scores of perceived fairness (ranging from 1 to 5), the higher the scores, the stronger sense of social justice.

#### Control variables

To reduce the potential estimation error caused by missing variables, we selected control variables based on the characteristics of older adults and their families collected through the survey. These variables included age, gender, ethnicity, residence, religious belief, marital status, education, individuals’ annual income in the last year, self-rated health, and the household economic status. The descriptive statistics of the main variables are shown in Table 1.

**Table 1:** Descriptive statistics of the main variables

Variables	Definitions	Mean/ Frequency	SD/ Percentage
Mental health	Frequency of depression: Always = 1; Often = 2; Sometimes = 3; Rarely = 4; Never = 5	3.734	1.027
SES perception	Lower = 1; Lower-middle = 2; Middle = 3; Upper-middle = 4; Upper = 5	2.176	0.900
Social class evaluation	Continuous variable: Ranging from 1 to 10	4.059	1.755
Social trust	Strongly disagree = 1; Relatively disagree = 2; Cannot say I agree or disagree = 3; Relatively agree = 4; Strongly agree = 5	3.631	0.970
Social justice	Totally unfair = 1; Relatively unfair = 2; Cannot say it is fair or unfair = 3; Relatively fair = 4; Totally fair = 5	3.294	1.052
Age	Continuous variable: Year	69.308	7.324
Gender	Male = 1;	2,069	51.15%
	Female = 0	1,976	48.85%
Residence	Urban = 1;	1,606	39.70%
	Rural = 0	2,439	60.30%
Ethnicity	Han = 1;	3,782	93.5%
	Otherwise = 0	263	6.5%
Education	Educated = 1;	3,105	76.76%
	Illiteracy = 0	940	23.24%
Income	Logarithm	8.116	3.542
Self-rated health	Very poor = 1; Poor = 2; Fair = 3; Good = 4; Very good = 5	2.984	1.075
Religious belief	Yes = 1;	470	11.62%
	No = 0	3,575	88.38%
Marital status	Married with a spouse = 1;	2,898	71.64%
	Otherwise = 0	1,147	28.36%
Household economic status	Much lower than average = 1; Lower than average = 2; Average = 3; Higher than average = 4; Much higher than average = 5	2.479	0.798

Note: For categorical variables, frequency and percent were reported; for continuous variables, means and standard deviation (S.D.) were reported.

### Analytic plan

In this study, Stata 14.0 was used for analysis.First, the ordinary least squares (OLS) regression was used to examine the relationship between SES perception and mental health. Meanwhile, the substitution methods of changing the model and independent variable were used to check the robustness of the basic results. Moreover, we used the Sobel model to analyse the mediating effects of social trust and justice in the relationship between SES perception and the mental health of older adults. Finally, we checked the relationship between the SES perception and mental health of Chinese older adults with the same objective SES to test the phenomenon of “*A contented mind is a perpetual feast*”.

## Results

### The relationship between SES perception and mental health

Table 2 represented the relationship between SES perception and the mental health of older adults. Column (1) suggested that SES perception was positively associated with the mental health of older adults (β = 0.072, *p* < 0.01). Column (2) reported the regression results by using Ordered Probit, and the result was consistent with Column (1). To further check the robustness of the results, we replaced the independent variable. The SES perception was replaced by social class evaluation. Columns (3) indicated that the results were consistent with the baseline results, which further indicated that the higher SES perception was related to better mental health among older adults.

In terms of controlled variables, increasing age and those older adults married with spouses were positively associated with mental health (β=0.007, *p*<0.01;β=0.092, *p*<0.01). The mental health of older adults from urban areas was significantly higher than those from rural areas (β= 0.177, *p*<0.01). In contrast, Han Chinese older adults and people with a religious belief were more likely to be depressed (β=-0.124, *p*<0.05;β=-0.096, *p*<0.05). Among education and health status, educated and better self-rated health were associated with better mental health (β = 0.104, *p*< 0.01; β = 0.350, *p*< 0.01). Finally, higher income and better household economic status contributed to better mental health (β = 0.022, *p*< 0.01; β = 0.096, *p*< 0.01). 

**Table 2:** Relationship between SES perception and mental health

	(1)	(2)	(3)
Variables	OLS	Ordered Probit	OLS
SES perception	0.072***	0.089***	
	(0.021)	(0.025)	
Social class evaluation			0.034***
			(0.010)
Age	0.007***	0.008***	0.006***
	(0.002)	(0.003)	(0.002)
Gender	0.036	0.047	0.036
	(0.030)	(0.036)	(0.030)
Residence	0.177***	0.236***	0.181***
	(0.034)	(0.041)	(0.034)
Ethnics	-0.124**	-0.145**	-0.125**
	(0.056)	(0.068)	(0.057)
Education	0.104***	0.123***	0.106***
	(0.037)	(0.043)	(0.037)
Income	0.022***	0.024***	0.022***
	(0.005)	(0.005)	(0.005)
Self-rated health	0.350***	0.413***	0.349***
	(0.014)	(0.018)	(0.014)
Religious belief	-0.096**	-0.110**	-0.094**
	(0.046)	(0.055)	(0.046)
Marital status	0.092***	0.110***	0.091***
	(0.033)	(0.040)	(0.033)
Household economic status	0.096***	0.110***	0.107***
	(0.024)	(0.029)	(0.022)
Constant	1.554***		1.558***
	(0.172)		(0.172)
Observations	4,045	4,045	4,045
R-squared/Pseudo R2	0.247	0.1000	0.247

Notes: * 0.1 ** 0.05 *** 0.01; Standard Errors in parentheses.

### Mediating effects of social trust and justice

**Fig. 1:**
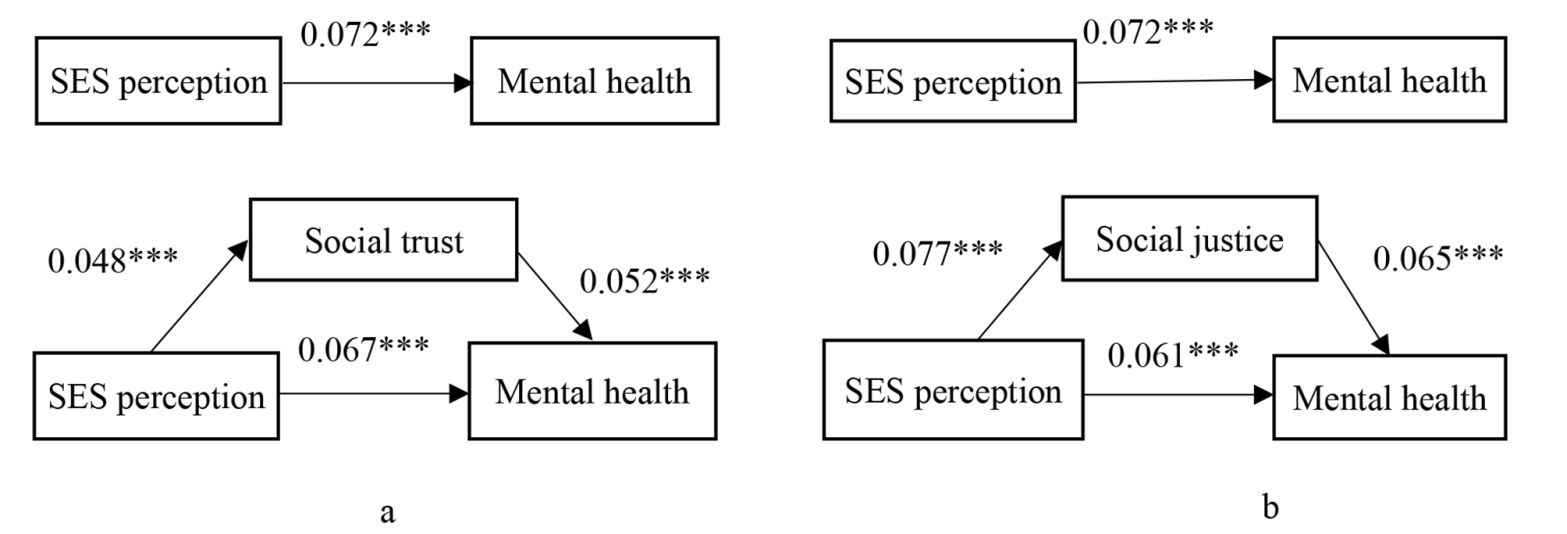
The mediating effects analysis

We used the Sobel model to examine the mediating effects of social trust and justice in the relationship between SES perception and mental health. Figure 1a showed the mediating results of social trust. The results showed that SES perception was positively associated with social trust (β = 0.048, *p* < 0.01), and social trust was positively related to mental health (β = 0.052, *p* < 0.01). When we controlled social trust, the correlation coefficient between SES perception and mental health decreased from 0.072 to 0.067 (*p* < 0.01), which indicated that social trust played a mediating role. The Sobel model showed the mediating proportion of the total effect was 6.44%.

Figure 1b showed that the mediating results of the relationship between SES perception, social justice, and mental health. The results showed that SES perception was significantly related tosocial justice (β = 0.077, *p* < 0.01), and social justice was positively associated with mental health (β = 0.065, *p* < 0.01). The correlation coefficient between SES perception and mental health decreased from 0.072 to 0.061 (*p* < 0.01) when we controlled social justice, which also indicated that the sense of social justice played a mediating role. The Sobel model showed the mediating proportion of the total effect was 15.18%.

### A contented mind is a perpetual feast: the relationship between SES perception and the mental health of older adults under the same objective SES

We divided the older adults into different groups based on objective SES. Previous studies used income, education attainment, and occupation as essential measures of objective SES [24, 65]. Considering that the participants were retirees, we did not use occupation as an objective SES indicator. Conversely, residence (urban and rural) is one of the main indicators of SES in China [19]. Therefore, we adopted the combination of income (high and low), education attainment (educated and illiterate), and residence (urban and rural) as indicators of objective SES. Specifically, we generated a new variable as an objective indicator of SES. The indicator was obtained by summing income, education, and residence; then, we obtained a score ranging from 0 to 3. The higher the score, the higher the objective SES of older adults.

Column (1) in Table 3 showed that the higher SES perception was associated with better mental health in the lowest objective SES group (β = 0.145, *P* < 0.05). Similarly, Column (4) in Table 3 also showed that higher SES perception was positively related to better mental health among the highest objective SES group (β = 0.122, *P* < 0.01). However, the positive relationship between SES perception and mental health was not significant among the other two groups of older adults.

**Table 3:** Tests of the relationship between SES perception and the mental health of older adults with different objective SES

Variables	(1) Objective SES = 0	(2) Objective SES = 1	(3) Objective SES = 2	(4) Objective SES = 3
SES perception	0.145**	0.031	0.033	0.122***
	(0.057)	(0.040)	(0.039)	(0.038)
Control variables	Yes	Yes	Yes	Yes
Constant	0.907**	1.852***	1.747***	2.396***
	(0.408)	(0.358)	(0.346)	(0.296)
Observations	527	1,039	1,074	1,405
R-squared	0.239	0.217	0.159	0.151

Notes: * 0.1 ** 0.05 *** 0.01; standard errors in parentheses; due to space limitations, we did not report the results of control variables.

## Discussion

Previous studies have mainly analysed the association between objective SES and people’s mental health, and the role of subjective SES has been neglected for a long time. Using data from the 2017 CGSS, this study examined the relationship between SES perception and mental health among older adults in China. First, the analysis revealed a significant positive correlation between SES perception and mental health, thereby supporting hypothesis 1. This finding aligns with previous research conducted by Adler et al. (2000) and Demakakos et al. (2008). Specifically, it was found that Chinese older adults experience better mental health when they have higher levels of SES perception. This can be attributed to the fact that older adults with low SES perception may face greater financial stress, which in turn contributes to poor mental health.

Moreover, we verified hypotheses 2 and 3, which postulated that the relationship between SES perception and mental health was mediated by socialtrust and justice. We proposed that SES perception affects individuals’ views on society, particularly in relation to social trust and justice, which form the core of an individual’s attitude towards society. On one hand, individuals with high levels of SES perceptions are more likely to succeed, which can lead to trust or inclination [66]. On the other hand, as a key aspect of social justice, equity in the distribution of power, resources, and processes ensures the sufficiency of the social determinants of health [67]. Therefore, individuals with low SES perceptions may experience feelings of deprivation, thereby exacerbating the sense of social injustice. Lack of social trust and justice may cause negative emotions and ultimately damage mental health.

Finally, we examined the phenomenon that *“a contented mind is a perpetual feast*” in Chinese society. In other words, this study analysed the relationship between SES perception and mental health among different groups based on the same objective SES. The results showed that SES perception did not always coincide with objective social status. The higher SES perception was related to better mental health when the objective SES remains the same. This is generally consistent with the findings of existing studies, which suggested that higher SES perception could still promote individuals’ wellbeing and health even when considering people’s objective social position, whereas low perceived SES has the opposite effect [25].

Additionally, the positive relationship between SES perception and mental health was significant for older adults with the lowest or highest SES. For individuals with the lowest SES, the increase in SES perception may reduce feelings of stress. The main reason remains that perceiving oneself as belonging to a lower subjective class serves as a source of stress itself [36]. In settings of the lowest SES, it may be more advantageous to manage emotions effectively, such as through cognitive reappraisal [68].

Correspondingly, older adults with high objective SES have more resources than others [22]. However, a limited socialization range prevents individuals from subjectively developing a reasonable SES perception. According to the social comparison theory, people assess their SES by comparing themselves to others [69]. Being surrounded by high SES people may lead to an underestimated SES perception, which is harmful to mental health [44]. In conclusion, the quote “*a contented mind is a perpetual feast*” persists in older Chinese adults, revealing how SES perceptions contribute to widening mental health disparities. Therefore, it is increasingly essential to improve the ability of SES perception, especially for those with the lowest or highest objective SES.

This study has several limitations. First, mental health was measured subjectively based on how often individuals experienced feelings of depression or frustration in the past four weeks. This subjective indicator could not comprehensively reflect the mental health status of older adults. In future studies, it is crucial to use more objective and comprehensive measures of mental health. Additionally, due to the cross-sectional nature of the CGSS data, our study could only reflect the correlation between the independent and dependent variables and could not identify causal effects. Finally, adverse causality between social trust or justice and mental health may occur in our analysis [70, 71]. However, due to data limitations, we failed to resolve this potential endogenous problem. We will conduct relevant research once panel data is available.

## Conclusions

Overall, this article highlights the significance of SES perception in enhancing the mental health of older adults and confirms the phenomenon that “*a contented mind is a perpetual feast*.” Our findings are helpful in identifying the relationship between SES perception and the mental health of older adults, as well as determining which kind of older adults are most in need of enhanced SES perception. This information can be used to provide tailored interventions to improve the mental health of older adults. In summary, enhancing SES perception not only improves the mental health of older persons directly but also indirectly through social trust and justice. In order to respond to population ageing, boosting SES perception is crucial for the overall well-being of older adults which contributes to ‘a contented mind’. The Chinese government could help older adults develop positive societal attitudes, particularly their social trust and justice, by creating a friendly environment.

## Data Availability

The datasets used and/or analysed during the current study are available from http://cgss.ruc.edu.cn/.
